# P-968. Academic - Practice Partnership to Advance Infection Prevention Education and Practice

**DOI:** 10.1093/ofid/ofae631.1158

**Published:** 2025-01-29

**Authors:** Mary Lou Manning, Kelly Zabriskie, John Renzi, Cindy M Hou

**Affiliations:** Thomas Jefferson University, College of Nursing, Conshohocken, Pennsylvania; Jefferson Health, Philadelphia, Pennsylvania; Jefferson Health, Philadelphia, Pennsylvania; Jefferson Health New Jersey, Startford, NJ

## Abstract

**Background:**

Nurses entering the workforce must have the requisite knowledge, skills, and abilities to recognize, prevent, and manage infection in individuals and populations, and be prepared to respond collaboratively to local, regional, and global infection prevention and control (IPC) challenges. Studies indicate that IPC content is lacking in US baccalaureate nursing (BSN) programs and graduates feel ill-prepared to implement infection risk mitigation strategies to protect patients and health care workers. Establishing academic- practice partnerships between colleges of nursing and health system IPC programs could close this gap.

**Methods:**

Our urban college of nursing, enrolling over 600 BSN students annually, convened a one-day invitational workshop focused on establishing an academic- practice partnership with our 18-hospital University-Health System IPC program. The workshop included presentations and small group work among expert inter-professional participants from IPC, nursing faculty and students, health system nursing, public health, and research.

**Results:**

A strategy and research agenda was developed to advance BSN student IPC education and practice, including opportunities for infection preventionists to inform academic content and provide IPC student clinical practice experiences. As an immediate outcome, we conducted a survey of our graduating BSN seniors (n=345) to gauge perceived value of a clinical IPC rotation, and a survey of student’s clinical preceptors (n=345) to elicit perceived ability of students to transfer their classroom knowledge into clinical practice. A quarter of the 86 student respondents (25%) expressed value in doing a clinical rotation in the IPC department. Almost 100% of the 160 preceptor respondents (46%) indicated that students successfully adapt classroom knowledge to various clinical practice situations.

**Conclusion:**

The workshop provided a forum to explore and begin to establish an academic- practice partnership between our college of nursing and the University-Health System IPC program. The survey results provide informative stakeholder data. The aim of this abstract is to report on the workshop proceedings, including short and long-term strategies, results of student and clinical preceptor surveys and next steps.

**Disclosures:**

**All Authors**: No reported disclosures

